# Supplementary material for: Prevalence of undiagnosed diabetes mellitus and associated factors among adult residents of Mizan Aman town, Southwest Ethiopia: Community-based cross-sectional study
Source: PLoS One. 2024 May 7;19(5):e0302167. doi: 10.1371/journal.pone.0302167 (PMC11075875; doi:10.1371/journal.pone.0302167)
Supplement: S1 File — (DOCX) [file pone.0302167.s002.docx]

## Amharic version

***በመረጃ የተደገፈ የስምምነት ወረቀት***

***በደቡብ ምዕራብ ኢትዮጵያ የሚዛን አማን ከተማ ጎልማሳ ነዋሪዎች ላይ ያልተመረመረ የስኳር በሽታ ሰርጭት እና ተያያዥ ምክንያቶችን ለመገምገም የተዘጋጀ መጠይቅ፤***

**የተሳታፊው መረጃ መቀበያ እና በፈቃደኝነት ላይ የተመሠረተ ስምምነት ቅፅ፤**

የኔ ስም __________ይባላል፡፡ በጅማ ዩኒቨርሲቲ የኤፒዲሚዮሎጂ ትምህርት ክፍል ተማሪ በሆነው በአቶ ፀጋዬ አትርሴ በአሁኑ ወቅት እየተካሄደ ላለዉ ጥናት መረጃ ሰብሳቢ ሆኜ እየሰራሁ ነው።

**የጥናቱ ርዕስ:**

ያልተመረመረ የስኳር በሽታ ስርጭት እና ተያያዥ ምክንያቶች።

**የጥናቱ ዓላማ:**

በሚዛን አማን ከተማ ነዋሪ ጎልማሶች ያልተመረመረ የስኳር በሽታ መጠን እና ተዛማጅ ምክንያቶች በመለየት ለመንግስት እና ለሌሎች ባለድርሻ አካላት ጠቃሚ መረጃ ለመስጠት፤ በሽታዉን ለመከላከል እና ለመቆጣጠር የሚያስችሉ ተግባራትን ለማቀድ።ለእኔ የምትሰጠኝ መረጃ ሥር በሰደዱ በሽታዎች ላይ የሚስተዋሉ ችግሮችን ለመቅረፍ የሚረዳ ሲሆን በተለይ በሚዛን አማን እና በአጠቃላይ በሀገሪቱ ያለውን በሽታ መከላከል የሚቻልበትን ነጥብ ይጠቁማል።

**ሂደት እና ቆይታ፡**

መጠይቁን ተጠቅሜ ቃለ መጠይቅ አደርግልዎታለሁ እና ለዚህ ጥናት የሚረዱትን 98 ጥያቄዎች ለመመለስ አስፈላጊ መረጃዎችን በምታቀርቡልኝ ጊዜ መጠይቆቹን እሞላለሁ። ቃለ-መጠይቁ ግማሽ ሰዓት ያህል ይወስዳል፡፡ የእርስዎን ክብደት፣ ቁመት፣ የወገብ እና ዳሌ ዙሪያ፣ የደም ግፊት እና የደም ስኳር መጠን ይለካሉ።ስለዚህ ለቃለ መጠይቁ እና ለመለካት ጊዜያችሁን እንድትሰጡ በትህትና እጠይቃለሁ።

**ስጋት እና ጥቅም፡**

በዚህ ጥናት ውስጥ የመሳተፍ ጊዜያችሁ ጥቂት ደቂቃዎችን ብቻ ከመውሰድ በስተቀር ምንም አይነት አደጋ የለም። ለጥናቱ ተሳታፊዎች ምንም አይነት ቀጥተኛ ክፍያ አይኖርም፡፡ ነገር ግን የጥናቱ ውጤት የፕሮግራም አስተዳዳሪዎች እና ፖሊሲ አውጪዎች በሁሉም ደረጃዎች ያልታወቀ የስኳር በሽታ መዘዝን ለማሸነፍ ውጤታማ ስትራቴጂ ለመንደፍ ይረዳል ።ማንኛውም ያልተለመደ የደም ግፊት እና የደም ስኳር መጠን የሕክምና ክትትል የሚያስፈልገው በአቅራቢያው ወደሚገኝ የጤና ተቋም ይላካል።

**ምስጢራዊነት፡** ያቀረቡት መረጃ ሚስጥራዊ ይሆናል። የእርስዎን መለያ የሚለይ መረጃ አይኖርም። የጥናቱ ግኝቶች ለማህበረሰብ አጠቃላይ ይሆናል እናም የግለሰብን ወይም የመኖሪያ ቤትን ምንም የሚያንፀባርቅ አይሆንም። ተሳታፊዎችን ከጥናቱ ጋር ሊያገናኙ የሚችሉ የቃል ወይም የጽሁፍ ዘገባዎች ማጣቀሻ አይደረግም።

**መብቶች፡**

የዚህ ጥናት ተሳትፎ በፈቃደኝነት ነው። በዚህ ጥናት ለመሳተፍ ወይም ላለመሳተፍ የማወጅ መብት አልዎትለመሳተፍ ከወሰኑ, በማንኛውም ጊዜ እና ከጥናቱ የመውጣት መብት አለዎት፡፡ጥናቱን ወይም አሰራሩን በሚመለከት በማንኛውም ጊዜ ጥያቄ ካሎት ለአቶ ፀጋዬ አትርሴ ሐል በስልክ -0949032098/0913427888 ደዉለው ያግኙ።

**በመረጃ ላይ የተመሰረተ የፈቃደኝነት ስምምነት መግለጫ፡-**

ከዚህ በታች በተገለፀው መሰረት ፊርማዬን በማኖር በዚህ ጥናት ለመሳተፍ በፈቃደኝነት መስማማቴን አውጃለሁ።

የተሳታፊው ፊርማ-------------------------- የመረጃ ሰብሳቢው ስም እና ፊርማ________________

የመረጃ አሰባሰብ ተቆጣጣሪ ስም እና ፊርማ ___________ ቀን፡- ________/________/2022

| Annex 4 Questionnaire (Amharic version) መጠይቅ | | | |  |
| --- | --- | --- | --- | --- |
| የዳሰሳ መረጃ | | | |  |
| ቦታ እና ቀን | | | ምላሽ | ኮድ |
|  | የቀበሌ ስም | | _________ | I2 |
|  | የመንደሩ ስም | |  |  |
|  | የጠያቂዉ ስም | | ____________ | I3 |
|  | መረጃዉ የተሞላበት ቀን | | ቀን----/ወር----/ዓ.ም--- | I4 |
| የተሳታፊ መታወቂያ ቁጥር ----- | | | | |
| ስምምነት፣ የቃለ መጠይቅ ቋንቋ እና ስም | | | ምላሽ | ኮድ |
|  | ፈቃድ ተገኝቷል? | | አዎ 2. አይደም | I5 |
|  | የቃለ መጠይቅ ቋንቋ | | ------------------- | I6 |
|  | የቃለ መጠይቁ ጊዜ (24 ሰዓት) | | ሰአት└─┴─┘: | I7 |
|  | ከተቻለ የስልክ ቁጥር ያግኙ | | __________ | I8 |
| **አንኳር፡ የስነሕዝብ መረጃ** | | | | |
|  | ፆታ | | 1. ወንድ 2. ሴት | C1 |
|  | እድሜዎ ስንት ነው? | | └─┴─┘ | C2 |
|  | በአጠቃላይ፣ በትምህርት ቤት ወይም በሙሉ ጊዜ ጥናት ምን ያህል አመታት አሳልፈዋል (ከቅድመ ትምህርት ቤት በስተቀር)? | | አመት/ታት **└─┴─┘** | C3 |
| **የሰፋ፡የሕዝብ መረጃ** | | | | |
|  | ያጠናቀቁት ከፍተኛ የትምህርት ደረጃ ምንድነው? | 1. መደበኛ ትምህርት ያልተማረ  2. ከአንደኛ ደረጃ ትምህርት ያላጠናቀቀ  3. የመጀመሪያ ደረጃ ትምህርት ያጠናቀቀ  4. ሁለተኛ ደረጃ ትምህርት ቤት ያጠናቀቀ  5. ሁለተኛ ደረጃ ትምህርት ቤት ያጠናቀቀ  6. ኮሌጅ/ ያጠናቀቀ 7. የድህረ ምረቃ ዲግሪ | | C4 |
|  | ብሄረሰብዎ ምንድነው? | 1.ቤንች 2.ካፋ3.ኦሮሞ4.አማራ 5. ትግሬ 6. ስልጤ  7.ጉራጌ 8. ሌላ | | C5 |
|  | ሃይማኖትዎ ምንድን ነው | 1. ፕሮቴስታንት 2. ኦርቶዶክስ 3. ሙስሊም 4. ካቶሊክ 5. ሌላ | |  |
|  | የጋብቻ ሁኔታ | 1.ያላገባ 2.ያገባ 3. የፈታ 4. አጋሩን በሞት ያጣ | | C6 |
|  | ከሚከተሉት ውስጥ ባለፉት 12 ወራት ውስጥ የእርስዎን ዋና የስራ ሁኔታ በተሻለ ሁኔታ የሚገልጸው የትኛው ነው? | 1. የመንግስት ሰራተኛ 4. ገበሬ 2. መንግስት ሰራተኛ ያልሆነ 5. ስራ የለለዉ 3. ነጋዴ 6.ተማሪ እምቢ 99 | | C7 |
|  | በእርስዎ ቤተሰብ ውስጥ ስንት ከ18 ዓመት በላይ የሆኑ ሰዎች ይኖራሉ?  (ራስን ጨምሮ) | የሰዉ ብዛት └─┴─┘ | | C8 |
|  | ጠቅላላ ዓመታዊ ገቢዎ በኢትዮጵያ ብር ስንት ነው።  (አንዱን ብቻ ይምረጡ | በዓመት └─┴─┴─┴─┴─┴─┴─┘ወደ T1 ሂድ | | C9a |
|  |  | በወር └─┴─┴─┴─┴─┴─┴─┘ወደ T1 ሂድ | | C9b |
|  |  | በሳምንት └─┴─┴─┴─┴─┴─┴─┘ወደ T1 ሂድ | | C9c |
|  |  | አላውቅም 77 | |  |
|  |  | እምቢ 99 | | C9d |

| **ደረጃ 1:የባህሪ መለኪያዎች**  የትምባሆ አጠቃቀም | | | | | | | | | | | | | |
| --- | --- | --- | --- | --- | --- | --- | --- | --- | --- | --- | --- | --- | --- |
| አሁን ስለተለያዩ የጤና ነክ ባህሪያት አንዳንድ ጥያቄዎችን ልጠይቅህ ነው። ይህም እንደ ማጨስ፣ አልኮል መጠጣት፣ አትክልትና ፍራፍሬ መመገብ እና የአካል ብቃት እንቅስቃሴን ይጨምራል። በትምባሆ እንጀምር። | | | | | | | | | | | | | |
|  | | በአሁኑ ጊዜ እንደ ሲጋራ ወይም የትምባሆ ምርቶችን ያጨሳሉ ? | | 1.አዎ 2. አይደለም አይደለም ከሆነ , ወደ ጥያቄ T6 ይሂዱ | | | | | | | | T1 | |
|  | | በአሁኑ ጊዜ የትምባሆ ምርቶችን በየቀኑ ያጨሳሉ? | | 1. አዎ 2. አይደለም ፤ አይደለም ከሆነ፣ T6 | | | | | | | | T2 | |
|  | | ለመጀመሪያ ጊዜ በየቀኑ ማጨስ የጀመርክበት ዕድሜህ ስንት ነበር ? | | ዕድሜ (በዓመት) └─┴─┘  አላዉቅም 77 ካወቀ, ወደ T5a ይሂዱ | | | | | | | | T3 | |
|  | | ምን ያህል ጊዜ እንደነበረ ታስታውሳለህ ? | | በዓመት └─┴─┘  ወይም በወር └─┴─┘  ወይም በሳምንት └─┴─┘ | | | | | | | | T4a  T4b  T4c | |
|  | | በአማካይ፣ ከሚከተሉት ውስጥ ምን ያህል በየቀኑ ያጨሳሉ?  ( ዕለታዊ አጫሾች ብቻ)  አላውቅም *77* | | የተሰሩ ሲጋራዎች └─┴─┘ | | | | | | | | T5a | |
|  |  |  |  | በእጅ የሚጠቀለል ሲጋራ └──┘ | | | | | | | | T5b | |
|  |  |  |  | በትምባሆ የተሞሉ ቱቦዎች └─┴─┘ | | | | | | | | T5c | |
|  |  |  |  | ሌላ └─┴─┘ ሌላ ከሆነ ወደ T5other ይሂዱ | | | | | | | | T5e | |
|  |  |  |  | አለበለዚያ ወደ T9 ይሂዱ | | | | | | | | T5other | |
| **የተስፋፋ፡ የትምባሆ አጠቃቀም** | | | | | | | | | | | | | |
|  | | ቀደም ባሉት ጊዜያት በየቀኑ ያጨሱ ነበር? | | | 1. አዎ 2. አይደለም ካልሆነ ወደ T9 ይሂዱ | | | | | | | T6 | |
|  | | በየቀኑ ማጨስ ስታቆም ዕድሜህ ስንት ነበር? | | | ዕድሜ (አመታት)└─┴─┘  አላውቅም ፤ የሚታወቅ ከሆነ ወደ T9 ይሂዱ | | | | | | | T7 | |
|  | | ምን ያህል ጊዜ እንደነበረ ታስታውሳለህ? (1 ብቻ፣ ሁሉም 3 አይደሉም)  አላውቅም 77 | | | በአመታት └─┴─┘ የሚታወቅ ከሆነ ወደ T9 ይሂዱ ወይም በወር ውስጥ └─┴─┘ የሚታወቅ ከሆነ ወደ T9 ይሂዱ ወይም በሳምንታት ውስጥ └─┴─┘ | | | | | | | T8a  T8b  T8c | |
|  | | በአሁኑ ጊዜ ማንኛውንም ትጠቀማለህ?ጭስ የሌለው ትንባሆ እንደ ማሽተት፣ ማኘክ ቢትል? | | | 1. አዎ 2. አይደለም ካልሆነ ወደ T12 ይሂዱ | | | | | | | T9 | |
|  | | በአሁኑ ጊዜ ጭስ የሌላቸው የትምባሆ ምርቶችን በየቀኑ ይጠቀማሉ? | | | 1. አዎ 2. አይደለም ካልሆነ ወደ T12 ይሂዱ | | | | | | | T10 | |
|  | | በአማካይ፣ በቀን ስንት ጊዜ ይጠቀማሉ….  (አላውቀውም 77 | | | ማሽተት፣ በአፍ └─┴─┘  ማሽተት፣ በአፍንጫ └─┴─┘  ትምባሆ ማኘክ └─┴─┘  ቤቴል፣ ኩይድ └─┴─┘  ሌላ └─┴─┘ | | | | | | | T11a  T11b  T11c  T11d  T11e  T11o | |
|  | | ቀደም ባሉት ጊዜያት ጭስ የሌለው ትንባሆ በየቀኑ እንደ ማሽተት፣ ትምባሆ ማኘክ ወይም ቢትል ተጠቅመህ ታውቃለህ? | | | 1. አዎ 2. አይደለም | | | | | | | T12 | |
|  | | ባለፉት 7 ቀናት ውስጥ፣ እርስዎ በሚኖሩበት ጊዜ በቤትዎ ውስጥ ያለ ሰው ያጨሰው በስንት ቀናት ነው? | | | የቀናት ብዛት └─┴─┘  አላውቅም 77 | | | | | | | T13 | |
|  | | ባለፉት 7 ቀናት ውስጥ እርስዎ በተገኙበት በስራ ቦታዎ ውስጥ አንድ ሰው በተዘጉ ቦታዎች ውስጥ ስንት ቀናት አጨስ? (በህንፃው ውስጥ፣ በስራ ቦታ ወይም በአንድ የተወሰነ ቢሮ ውስጥ) | | | የቀናት ብዛት └─┴─┘  በተዘጋ ቦታ ላይ አታውቅም አልሰራም 77 | | | | | | | T14 | |
| **የአልኮል አጠቃቀም** | | | | | | | | | | | | | |
| የሚቀጥሉት ጥያቄዎች ስለ አልኮል መጠጥ ይጠይቃሉ | | | | | | | | | | | | | |
|  | | እንደ ቢራ፣ ወይን፣ መናፍስት፣ የፈላ ሲደር ያሉ የአልኮል መጠጦችን ጠጥተህ ታውቃለህ? | | | 1. አዎ 2. አይደለም 2 ከሆነ፣ ወደ D1 ይሂዱ | | | | | | | A1a | |
|  | | ባለፉት 12 ወራት ውስጥ የአልኮል መጠጥ ጠጥተዋል? | | | 1. አዎ 2.አይደለም 2 ከሆነ፣ ወደ D1 ይሂዱ | | | | | | | A1b | |
|  | | ባለፉት 12 ወራት ውስጥ ቢያንስ አንድ የአልኮል መጠጥ ምን ያህል በተደጋጋሚ ጠጥተዋል?? | | | 1. በየቀኑ 2. በሳምንት 5-6 ቀናት  3. በሳምንት 1-4 ቀናት 4. በሳምንት 1-3 ቀናት  5. በወር ከአንድ ጊዜ ያነሰ | | | | | | | A2 | |
|  | | ባለፉት 30 ቀናት ውስጥ የአልኮል መጠጥ ጠጥተዋል? | | | 1. አዎ 2. አይደለም 2 ከሆነ፣ ወደ D1 ይሂዱ | | | | | | | A3 | |
|  | | ባለፉት 30 ቀናት ውስጥ፣ ስንት አጋጣሚዎች አሎት? | | | ቁጥር└─┴─┘ አላውቅም 77 | | | | | | | A4 | |
|  | | ባለፉት 30 ቀናት ውስጥ፣ አልኮል ሲጠጡ፣በአማካኝ፣በአንድ የመጠጥ ወቅት ምን ያህል መደበኛ የአልኮል መጠጦች ነበራችሁ? ┘ | | | ቁጥር└─┴─┘  አላውቅም 77 | | | | | | | A5 | |
|  | | በአለፉት 30 ቀናት ውስጥ ሁሉንም አይነት የአልኮል መጠጦችን አንድ ላይ በመቁጠር በአንድ ጊዜ ከጠጡት መደበኛ የአልኮል መጠጦች ትልቁ ምን ያህል ነበር? | | | ትልቁ ቁጥር └─┴─┘  አላውቅም 77 | | | | | | | A6 | |
|  | | ባለፉት 30 ቀናት ውስጥ ምን ያህል ጊዜ አሎት፡-ለወንዶች አምስት ወይም ከዚያ በላይ  ለሴቶች፡ አራት ወይም ከዚያ በላይ መደበኛ የአልኮል መጠጦች በአንድ ጊዜ መጠጣት? | | | ቁጥር└─┴─┘  አላውቅም 77 | | | | | | | A7 | |
| **አልኮል የቀጠለ** | | | | | | | | | | | | | |
|  | | ባለፉት 30 ቀናት ውስጥ፣ የአልኮል መጠጥ ሲጠጡ፣ ከምግብ ጋር ምን ያህል ጊዜ ነበር?(መክሰስ አይቁጠሩ።) | | | 1. ብዙውን ጊዜ ከምግብ ጋር 2. አንዳንድ ጊዜ ከምግብ ጋር 3. ከምግብ ጋር እምብዛም  4. በጭራሽ ከምግብ ጋር | | | | | | | A8 | |
|  | | በእያንዳንዳቸው 7 ቀናት ውስጥ፣ በየቀኑ ምን ያህል መደበኛ የማንኛውም የአልኮል መጠጥ ይጠጡ ነበር? | | | ሰኞ └─┴─┘ | | | | | | | A9a | |
|  |  |  |  |  | ማክሰኞ └─┴─┘ | | | | | | | A9b | |
|  |  |  |  |  | እሮብ └─┴─┘ | | | | | | | A9c | |
|  |  |  |  |  | ሐሙስ └─┴─┘ | | | | | | | A8d | |
|  |  |  |  |  | አርብ └─┴─┘ | | | | | | | A9e | |
|  |  |  |  |  | ቅዳሜ └─┴─┘ | | | | | | | A9f | |
|  |  |  |  |  | እሁድ └─┴─┘ | | | | | | | A9g | |
| **ጫት መቃም** | | | | | | | | | | | | | |
|  | | ጫት ቅመህ ተውቃለህ/ታዉቂያለሽ? | | | 1.አዎ 2.አይደለም ፤ አይደለም ከሆነ ወደ D1 ሂድ | | | | | | | K1 | |
|  | | በአሁኑ ጊዜ ጫት ትቅማለህ/ትቅምያለሽ? | | | 1.አዎ 2.አይደለም ፤ አይደለም ከሆነ ወደ D1 ሂድ | | | | | | | K2 | |
|  | | ባለፉት 12 ወራት በየስንት ጊዜ ቅመህ ታውቃለህ/ታዉቂያለሽ? | | | 1. በየቀኑ  2. 5-6 ጊዜ ሳምንት  3. 3-4 ጊዜ ሳምንት  4. 1-2 ጊዜ ሳምንት  5. 1-3 ጊዜ በወር  6. በወር ከ 1 ጊዜ በታች | | | | | | | K3a  K3b  K3c  K3d  K3e  K3f | |
|  | | መጀመሪያ መቃም ስትጀምር ዕድሜህ/ሽ ስንት ነበር? | | | --------------------------------------------------- | | | | | | | K4 | |
|  | | ስንት ዓመት እንደሚሆን ታስታዉሳለህ/ታስታዉሻለሽ? | | | 1.በዓመት----------------  2.በወር------------------  በሳምንት--------------- | | | | | | | K5a  K5b  K5c | |
|  | | በአማካይ ስንት ዞርባ በሳምንት ወይም በቀን ትቅማለህ/ሽ? | | | ------------------------ | | | | | | | K6 | |
|  | | ባለፉት 12 ወራት ጫት መቃም ለማቆም ሞክረሃል? | | | 1.አዎ 2. አይደለም | | | | | | | K7 | |
| **ኮር: አመጋገብ** | | | | | | | | | | | | | |
| የሚቀጥሉት ጥያቄዎች ብዙውን ጊዜ ስለሚበሉት አትክልትና ፍራፍሬ ይጠይቃሉ። እዚህ የአገሬው አትክልትና ፍራፍሬ ምሳሌዎችን የሚያሳይ የአመጋገብ ካርድ አለኝ። እያንዳንዱ ሥዕል የአገልግሎቱን መጠን ይወክላል። ለእነዚህ ጥያቄዎች መልስ ስትሰጥ እባክህ ያለፈውን አመት የተለመደ ሳምንት አስብ | | | | | | | | | | | | | |
|  | | በተለመደው ሳምንት ውስጥ ስንት ቀናት ፍራፍሬ ይበላሉ? | | | ቁጥር └─┴─┘ ዜሮ ከሆነ ወደ D3 ይሂዱ | | | | | | | D1 | |
|  | | ከእነዚያ ቀናት በአንዱ ስንት ፍሬ ይበላሉ? | | | የመመገቢያ ብዛት └─┴─┘ | | | | | | | D2 | |
|  | | በተለመደው ሳምንት ውስጥ ስንት ቀናት አትክልቶችን ይበላሉ? | | | የቁጥር ቀናት └─┴─┘  ዜሮ ከሆነ ወደ D5 ይሂዱ | | | | | | | D3 | |
|  | | ከእነዚያ ቀናት በአንዱ ስንት አትክልት ይበላሉ? | | | የቁጥር አቅርቦቶች └─┴─┘ | | | | | | | D4 | |
| **የቀጠለ** | | | | | | | | | | | | | |
|  | በቤተሰብዎ ውስጥ ለምግብ ዝግጅት ምን ዓይነት ዘይት ወይም ቅባት በብዛት ጥቅም ላይ ይውላል?  አላውቅም 77 | | | | 1.የአትክልትዘይት2.ቅቤ 3.ሻኖ 4.ማርጋሪን 5. የተለየ አይደለም 6. ምንም ጥቅም ላይ አልዋለም  7ጋርሊጊ 8.ሌላ ከሆነ ወደ D5ሌላ ይሂዱ | | | | | | | D5 | |
|  | በአማካይ፣ በቤት ውስጥ ያልተዘጋጁ በሳምንት ስንት ምግቦች ይበላሉ? ? | | | | የቁጥር አቅርቦቶች └─┴─┘  አላውቅም 77 | | | | | | | D6 | |
| ኮር: አካላዊ እንቅስቃሴ | | | | | | | | | | | | | |
| በመቀጠል በተለመደው ሳምንት ውስጥ የተለያዩ አይነት የአካል ብቃት እንቅስቃሴዎችን በማድረግ የምታሳልፈውን ጊዜ ልጠይቅህ ነው። እባክህ እራስህን እንደ አካላዊ ንቁ ሰው ባትቆጥርም እንኳን እነዚህን ጥያቄዎች መልስ። በመጀመሪያ ስራ ለመስራት ስለሚያሳልፉት ጊዜ ያስቡ. ሥራን እንደ የሚከፈል ወይም ያልተከፈለ ሥራ፣ ጥናት/ሥልጠና፣ የቤት ውስጥ ሥራዎችን፣ ምግብ/ሰብሎችን መሰብሰብ፣ ዓሣ ማጥመድ ወይም ምግብ ማደን፣ ሥራ መፈለግን የመሳሰሉ ማድረግ ያለብዎትን ነገሮች አድርገው ያስቡ። | | | | | | | | | | | | | |
|  | | ስራዎ ቢያንስ ለ10 ደቂቃ ያለማቋረጥ ከፍተኛ የአተነፋፈስ ወይም የልብ ምት እንዲጨምር የሚያደርገውን የጠንካራ-ጥንካሬ እንቅስቃሴን ያካትታል? (ከባድ ሸክሞችን መሸከም ወይም ማንሳት፣መቆፈር ወይም የግንባታ ስራ) | | | | 1. አዎ 2. አይደለም ፤ ካልሆነ ወደ P4 ይሂዱ | | | | | | | P1 |
|  | | በተለመደው ሳምንት ውስጥ፣ በስንት ቀናት ውስጥ የጠንካራ ጥንካሬ እንቅስቃሴዎችን እንደ የስራዎ አካል ያደርጋሉ? | | | | የቀናት ብዛት└─┘ | | | | | | | P2 |
|  | | በተለመደው ቀን በሥራ ቦታ ብርቱ-ጥንካሬ እንቅስቃሴዎችን ለማድረግ ምን ያህል ጊዜ ታጠፋለህ? በቀላሉ ለማስታወስ አንድ ቀን አስብ፡፡ለ10 ደቂቃ ወይም ከዚያ በላይ ያለማቋረጥ የተከናወኑ ተግባራትን ብቻ አስቡባቸው። ለማረጋገጥ በጣም ከፍተኛ ምላሾችን (ከ4 ሰአታት በላይ) ይፈትሹ። | | | | ሰዓታት: ደቂቃዎች  └─┴─┘: └─┴┘ | | | | | | | P3  (a-b) |
|  | | ስራዎ አነስተኛ የአተነፋፈስ መጨመርን የሚያስከትል መጠነኛ-ጥንካሬ እንቅስቃሴን ያካትታል ወይም የልብ ምት እንደ ቢያንስ ለ 10 በፍጥነት መራመድ ያለማቋረጥ ደቂቃዎች?  [ወይም ቀላል ሸክሞችን መሸከም] | | | | 1. አዎ  2. አይ  ካልሆነ ወደ P7 ይሂዱ | | | | | | | P4 |
|  | | በተለመደው ሳምንት ውስጥ እንደ የስራዎ አካል መጠነኛ የጥንካሬ እንቅስቃሴዎችን በስንት ቀናት ውስጥ ይሰራሉ? | | | | የቀናት ብዛት └─┘ | | | | | | | P5 |
|  | | በተለመደው ቀን በሥራ ቦታ መጠነኛ-ጥንካሬ እንቅስቃሴዎችን ለማድረግ ምን ያህል ጊዜ ታጠፋለህ? ለማረጋገጥ በጣም ከፍተኛ ምላሾችን (ከ4 ሰአታት በላይ) ይፈትሹ) | | | | ሰዓታት : ደቂቃ └─┴─┘: └─┴─┘  ሰዓት ደቂቃ | | | | | | | P6  (a-b) |
| **ወደ እና ከቦታ ወደ ቦታ መጓዝ** | | | | | | | | | | | | | |
| የሚቀጥሉት ጥያቄዎች ቀደም ሲል የጠቀስካቸውን በሥራ ላይ ያሉ አካላዊ እንቅስቃሴዎችን አያካትቱም።አሁን ወደ ቦታዎች እና ወደ ቦታው ስለምትጓዝበት የተለመደ መንገድ ልጠይቅህ እፈልጋለሁ። ለምሳሌ ለመሥራት፣ ለገበያ፣ ለገበያ፣ ለአምልኮ ቦታ። | | | | | | | | | | | | | |
|  | | ከቦታ ወደ ቦታ ለመሄድ እና ለመነሳት ያለማቋረጥ ቢያንስ ለ10 ደቂቃዎች በብስክሌት (ፔዳል ዑደት) ይራመዳሉ ወይም ይጠቀማሉ? | | | | 1. አዎ 2. አይደለም  ካልሆነ ወደ P10 ይሂዱ | | | | | | | P7 |
|  | | በተለመደው ሳምንት ውስጥ፣ ወደ ቦታዎች ለመሄድ እና ለመሄድ ያለማቋረጥ ቢያንስ ለ10 ደቂቃ በስንት ቀናት ውስጥ በእግር ወይም በብስክሌት ይራመዳሉ? | | | | የቀናት ብዛት└─┘ | | | | | | | P8 |
|  | | በተለመደው ቀን በእግር ወይም በብስክሌት መንዳት ምን ያህል ጊዜ ያሳልፋሉ? በጣም ከፍተኛ ምላሾችን ይመርምሩ /> 4hr | | | | ሰዓታት ደቂቃዎች  └─┴─┘: └─┴─┘  ሰዓት ደቂቃ | | | | | | | P9  (a-b) |
| **የመዝናኛ እንቅስቃሴዎች** | | | | | | | | | | | | | |
| የሚቀጥሉት ጥያቄዎች ቀደም ሲል የጠቀስካቸውን የሥራ እና የትራንስፖርት እንቅስቃሴዎች አያካትትም.አሁን ስለ ስፖርት፣ የአካል ብቃት እና የመዝናኛ እንቅስቃሴዎች ልጠይቅዎት እፈልጋለሁ። | | | | | | | | | | | | | |
|  | | እንደ (ሩጫ ወይም እግር ኳስ) ያለማቋረጥ ቢያንስ ለ10 ደቂቃ ያህል ከፍተኛ የአተነፋፈስ ወይም የልብ ምት እንዲጨምር የሚያደርጉ ጠንካራ-ጠንካራ ስፖርቶች፣ የአካል ብቃት ወይም የመዝናኛ (የመዝናኛ) እንቅስቃሴዎችን ያደርጋሉ? | | | | 1. አዎ 2. አይደለም  ካልሆነ ወደ P 13 ይሂዱ | | | | | | | P10 |
|  | | በተለመደው ሳምንት ውስጥ፣ በስንት ቀናት ውስጥ ኃይለኛ-ጥንካሬ ስፖርቶችን፣ የአካል ብቃት ወይም የመዝናኛ (የመዝናኛ) እንቅስቃሴዎችን ታደርጋለህ? | | | | የቀናት ብዛት └─┘? | | | | | | | P11 |
|  | | በተለመደው ቀን ኃይለኛ-ጠንካራ ስፖርቶችን፣ የአካል ብቃት ወይም የመዝናኛ እንቅስቃሴዎችን በማድረግ ምን ያህል ጊዜ ታጠፋለህ?(በጣም ከፍተኛ ምላሾችን መርምር (ከ4 ሰአታት በላይ) | | | | ሰዓት፡ -------- ደቂቃ ----------: | | | | | | | P12  (a-b) |
|  | | በትንሹ ለ10 ደቂቃ ያለማቋረጥ የትንፋሽ መጨመር ወይም የልብ ምት እንደ ፈጣን መራመድ፣ (ብስክሌት መንዳት፣ ዋና እና ቮሊቦል) የሚያስከትሉ መጠነኛ-ጥንካሬ ስፖርቶች፣ የአካል ብቃት ወይም የመዝናኛ እንቅስቃሴዎች ታደርጋለህ? | | | | 1. አዎ 2. አይደለም ካልሆነ ወደ P16 ይሂዱ | | | | | | | P13 |
|  | | በተለመደው ሳምንት ውስጥ፣ በስንት ቀናት ውስጥ መጠነኛ-ጥንካሬ ስፖርቶችን፣ የአካል ብቃት ወይም የመዝናኛ እንቅስቃሴዎችን ታደርጋለህ? | | | | የቀናት ብዛት └─┘ | | | | | | | P14 |
|  | | በተለመደው ቀን መጠነኛ-ጥንካሬ ስፖርቶችን፣ የአካል ብቃት ወይም የመዝናኛ እንቅስቃሴዎችን ለማድረግ ምን ያህል ጊዜ ታጠፋለህ? | | | | ሰዓት: ደቂቃ └─┴─┘: └─┴─┘  ሰዓት ደቂቃ | | | | | | | P15  (a-b) |
| **አካላዊ እንቅስቃሴ የቀጠለ** | | | | | | | | | | | | | |
| **ያለመንቀሳቀስ ባህሪ** | | | | | | | | | | | | | |
| የሚከተለው ጥያቄ በሥራ ቦታ፣ ቤት ውስጥ፣ ወይም ከጓደኞች ጋር በጠረጴዛ ዙሪያ ተቀምጠው ማዉራት፣ ፣ በመኪና፣ በአውቶቡስ፣ በባቡር፣ በማንበብ፣ በመጫወት ወይም ቴሌቪዥን በመመልከት ያሳለፉትን ጊዜ ጨምሮ ስለመቀመጥ ነው። ነገር ግን በእንቅልፍ ጊዜ የሚያሳልፉትን ጊዜ አያካትቱ፡፡ | | | | | | | | | | | | | |
|  | | በተለመደው ቀን ምን ያህል ጊዜ በመቀመጥ ወይም በመቀመጫ ያሳልፋሉ? | | | | ሰዓታት -----: ደቂቃ --------: | | | | | | | P16  (a-b) |
| ኮር: ከፍ ያለ የደም ግፊት ታሪክ | | | | | | | | | | | | | |
|  | የደም ግፊትዎን በሃኪም ወይም በሌላ የጤና ሰራተኛ ተለካህ ታውቃለህ? | | | | | 1.አዎ 2. አይደለም ፤ ካልሆነ ወደ H6 ይሂዱ | | | | | | | H1 |
|  | የደም ግፊትን ወይም የደም ግፊት መጨመርን በሃኪም ወይም በሌላ የጤና ሰራተኛ ተነግሮዎት ያውቃል? | | | | | 1. አዎ 2. አይደለም ፤ካልሆነ ወደ H6 ይሂዱ | | | | | | | H2a |
|  | ባለፉት 12 ወራት ውስጥ ተነግሯችኋል? | | | | | 1. አዎ 2. አይደለም | | | | | | | H2b |
| የደም ግፊት መጨመር ታሪክ የቀጠለ | | | | | | | | | | | | | |
|  | | በአሁኑ ጊዜ በሀኪም ወይም በሌላ የጤና ሰራተኛ ከታዘዙት ለከፍተኛ የደም ግፊት ህክምናዎች/ ምክሮች ከሚከተሉት ውስጥ አንዳቸውንም እየተቀበሉ ነው? | | | | | | | | | | | |
|  |  | ባለፉት ሁለት ሳምንታት ውስጥ የወሰዷቸው መድሃኒቶች (መድሃኒት) | | | | | | | | | | | H3a |
|  |  | የጨው መጠን ለመቀነስ ምክር | | | | | | | | | | | H3b |
|  |  | ክብደትን ለመቀነስ ምክር ወይም ህክምና | | | | | | | | | | | H3c |
|  |  | ማጨስን ለማቆም ምክር ወይም ህክምና | | | | | | | | | | | H3d |
|  |  | የአካል ብቃት እንቅስቃሴ ለመጀመር ወይም ተጨማሪ የአካል ብቃት እንቅስቃሴ ለማድረግ ምክር አግኝተዋል | | | | | | | | | | | H3e |
|  | | የደም ግፊት መጨመር ወይም ለደም ግፊት የባህል ሐኪም አማክረህ ታውቃለህ? | | | | 1. አዎ  2. አይ | | | | | | | H4 |
|  | | በአሁኑ ጊዜ ለደም ግፊትዎ ከዕፅዋት የተቀመሙ ወይም ባህላዊ መድኃኒት እየወሰዱ ነው? | | | | 1. አዎ  2. አይ | | | | | | | H5 |
| **ኮር: የስኳር በሽታ ታሪክ** | | | | | | | | | | | | | |
|  | | በደምዎ ውስጥ ያለውን የስኳር መጠን በዶክተር ወይም በሌላ የጤና ሰራተኛ ተለክተው ያውቃሉ? | | | | 1.አዎ 2. አይደለም ካልሆነ ወደ M1 ይሂዱ | | | | | | | H6 |
|  | | በደምዎ ውስጥ ያለውን የስኳር መጠን ወይም የስኳር በሽታ እንዳለብዎት በሃኪም ወይም በሌላ የጤና ሰራተኛ ተነግሮዎት ያውቃሉ? | | | | 1. አዎ 2. አይደለም አይ ካልሆነ ወደ M1 ይሂዱ | | | | | | | H7a |
|  | | ባለፉት 12 ወራት ውስጥ ተነግሯችኋል? | | | | 1. አዎ 2. አይደለም | | | | | | | H7b |
| **የስኳር በሽታ ታሪክ የቀጠለ** | | | | | | | | | | | | | |
|  | | በአሁኑ ጊዜ በሃኪም ወይም በሌላ የጤና ሰራተኛ ከታዘዙት የስኳር ህመም ህክምናዎች/ ምክሮች ውስጥ አንዱን እየተቀበሉ ነው? | | | | | | | | | | |  |
|  |  | ኢንሱሊን | | | | | | | 1. አዎ 2. አይደለም | | | | H8a |
|  |  | ባለፉት ሁለት ሳምንታት ውስጥ የወሰዷቸው መድሃኒቶች (መድሃኒት). | | | | | | | 1. አዎ 2. አይደለም | | | | H8b |
|  |  | ልዩ የታዘዘ አመጋገብ | | | | | | | 1. አዎ 2. አይደለም | | | | H8c |
|  |  | ክብደትን ለመቀነስ ምክር ወይም ህክምና | | | | | | | 1. አዎ 2. አይደለም | | | | H8d |
|  |  | ማጨስን ለማቆም ምክር ወይም ህክምና | | | | | | | 1. አዎ 2. አይደለም | | | | H8e |
|  |  | ተጨማሪ ልምምድ ለመጀመር ወይም ለመስራት ምክር | | | | | | | 1. አዎ 2. አይደለም | | | | H8f |
|  | | ለስኳር ህመም የባህል ሀኪም አማክረህ ታውቃለህ? | | | | | | | 1. አዎ 2. አይደለም | | | | H9 |
|  | | በአሁኑ ጊዜ ለስኳር ህመምዎ ከዕፅዋት የተቀመሙ ወይም ባህላዊ መድኃኒቶችን እየወሰዱ ነው? | | | | | | | 1. አዎ 2. አይደለም | | | | H10 |
|  | | በእርግዝና ወቅት እናትህ የእርግዝና የስኳር በሽታ ነበረባት? | | | | | | | 1. አዎ 2. አይደለም | | | | H11g |
|  | | ለስኳር ህመም የተረጋገጠ ወይም የስኳር ህመምተኛ ታሪክ ያለው የቤተሰብ አባል ወይም ዘመድ (አባት ፣ እናት ፣ እህት ፣ አያት ቤተሰብ ፣ ወዘተ.) አለ? | | | | | | | 1. አዎ 2. አይደለም | | | | H11 |
| **ደረጃ 2 አካላዊ መለኪያዎች** | | | | | | | | | | | | | |
| **ቁመት እና ክብደት** | | | | | | | | | | | | | |
|  | | የጠያቂ መታወቂያ(ለHt,wt &WC) | | | | | | | └─┴─┴─┘ | | | | M1 |
|  | | የመሳሪያ መታወቂያዎች (ቁመት እና ክብደት) | | | | | | | ቁመት └─┴─┘  ክብደት └─┴─┘ | | | | M2a  M2b |
|  | | ቁመት(የተሳታፊውን ቁመት በሴሜ መዝግብ) | | | | | | | በሴንቲሜትር (ሴሜ) └─┴─┴─┘.└─┘ | | | | M3 |
|  | | ክብደት (ለሚዛን በጣም ትልቅ ከሆነ ኮድ 666.6 | | | | | | | በኪሎግራም└─┴─┴─┘.└─┘ | | | | M4 |
|  | | ለሴቶች፡ ነፍሰ ጡር ነሽ? | | | | | | | 1. አዎ 2. አይደለም አዎ ከሆነ፣ ወደ M 8 ይሂዱ | | | | M5 |
| **ወገብ** | | | | | | | | | | | | | |
|  | | የወገብ መለኪያ መሣሪያ መታወቂያ | | | | | | | └─┴─┘ | | | | M6 |
|  | | የወገብ ዙሪያ ልኬት | | | | | | | በሴንቲሜትር└─┴─┴─┘.└─┘ | | | | M7 |
| **የደም ግፊት** | | | | | | | | | | | | | |
|  | | የጠያቂ መታወቂያ | | | | | | └─┴─┴─┘ | | | M8 | | |
|  | | የመሣሪያ መታወቂያ | | | | | | └─┴─┘ | | | M9 | | |
|  | | ጥቅም ላይ የዋለው Cuff መጠን | | | | | | 1. ትንሽ 2. መካከለኛ 3. ትልቅ | | | M10 | | |
|  | | ንባብ 1 | | | | | | ሲስቶሊክ (mmHg) └─┴─┴─┘  ዲያስቶሊክ (mmHg )└─┴─┴─┘ | | | M11a  M11b | | |
|  | | ንባብ 2  (ተሳታፊው ለ 15 ደቂቃዎች ካረፈ በኋላ የመጀመሪያውን መለኪያ ይመዝግቡ እና 3 ኛ መለኪያ ከመውሰዳቸው በፊት 3 ደቂቃዎች ይጠብቁ) | | | | | | ሲስቶሊክ (mmHg) └─┴─┴─┘  ዲያስቶሊክ (mmHg )└─┴─┴─┘ | | | M12a  M12b | | |
|  | | ንባብ 3 (ተሳታፊው ለ 15 ደቂቃዎች ካረፈ በኋላ የመጀመሪያውን መለኪያ  ይመዝግቡ) | | | | | | ሲስቶሊክ (mmHg) └─┴─┴─┘  ዲያስቶሊክ (mmHg )└─┴─┴─┘ | | | M13a  M13b | | |
|  | | ባለፉት ሁለት ሳምንታት ውስጥ ለደም ግፊት መጨመር በሀኪም ወይም በሌላ የጤና ሰራተኛ የታዘዙ መድሃኒቶች (መድሃኒት) ታክመዋል? | | | | | | 1. አዎ 2. አይደለም | | | M14 | | |
| **የቀጠሌ: የወገብ ዙሪያ እና የልብ ምት** | | | | | | | | | | | | | |
|  | | የወገብ ዙሪያ | በሴንቲሜትር (ሴሜ)└─┴─┴─┘.└─┘ | | | | | | | M15 | | | |
|  | | የልብ ምት (ሶስቱን የልብ ምት ንባቦችን ይመዝግቡ ) | | | | | | | | | | | |
|  |  | ማንበብ 1 | ምት በደቂቃ └─┴─┴─┘ | | | | | | | M16a | | | |
|  |  | ማንበብ 2 | ምት በደቂቃ └─┴─┴─┘ | | | | | | | M16b | | | |
|  |  | ማንበብ | ምት በደቂቃ └─┴─┴─┘ | | | | | | | M16c | | | |
| **ደረጃ 3 ባዮኬሚካል መለኪያዎች** | | | | | | | | | | | | | |
| **የደም ግሉኮስ** | | | | | | | | | | | | | |
|  | | ባለፉት 12 ሰአታት ውስጥ ከውሃ ውጪ የበሉት ወይም የጠጡት ነገር አልዎት? | | | | | 1.አዎ አይደለም | | | | B1 | | |
|  | | የቴክኒሽያን መታወቂያ | | | | | └─┴─┴─┘ | | | | B2 | | |
|  | | የመለኪያ መሳሪያ መታወቂያ | | | | | └─┴─┘ | | | | B3 | | |
|  | | የደም ናሙናዉ የተወሰደበት ሰዓት (24 ሰዓት) | | | | | ሰዓት : ደቂቃ └─┴─┘: └─┴─┘ | | | | B4 | | |
|  | | የተለካዉ የደም ግሉኮስ መጠን | | | | | ሚግ /ዴሊ └─┴─┘. └─┴─┘ | | | | B5 | | |
|  | | የደም ግሉኮስዎ በመጨመሩ ምክንያት ዛሬ በዶክተር ወይም ሌላ የጤና ሰራተኛ የታዘዙ ኢንሱሊን ወይም ሌሎች መድሃኒቶችን (መድሃኒት) ወስደዋል ወይም? | | | | | 1.አዎ አይደለም | | | | B6 | | |
