# Supplementary material for: Prevalence of undiagnosed diabetes mellitus and associated factors among adult residents of Mizan Aman town, Southwest Ethiopia: Community-based cross-sectional study
Source: PLoS One. 2024 May 7;19(5):e0302167. doi: 10.1371/journal.pone.0302167 (PMC11075875; doi:10.1371/journal.pone.0302167)
Supplement: S2 File — (DOCX) [file pone.0302167.s003.docx]

**ANNEXES**

**Annex 1: Informed consent sheet (English version)**

*Questionnaires to assess the magnitude of undiagnosed diabetes mellitus and its associated factors among adult residents of Mizan-Aman town, southwest Ethiopia*

**Participant’s information sheet and voluntary informed consent**

My Name is ________________________________. I am working as a data collector for
the study being conducted by Mr. Tsegaye Atrese, who is currently a student at Jimma University, department of Epidemiology.

**Title of the study:** Community-based study of the magnitude of undiagnosed diabetes mellitus and its associated factors among adult residents of Mizan-Aman town, southwest Ethiopia.
**Aim of the study:** To assess magnitude of undiagnosed diabetes mellitus and its associated factors among adult residents of Mizan-Aman town so as to provide important information to government and other stakeholders to plan for possible intervention activities on the disease under study. The information you provide me/us could help to overcome the problems related to chronic diseases and indicate the point where to prevent the disease in Mizan-Aman in particular and in the country at large.

**Procedure and duration:** I will be interviewing you using a questionnaire and I will fill the questionnaires while you are providing me pertinent data on questions to be answered which are helpful for this study. The interview will take about half an hour. Likewise, I will
measure your weight, height, waist circumference, blood pressure, and your blood sugar.
Therefore, I kindly demand you to devote your time for the interview and measurements.
**Risk and benefit:** There is no risk of being participating in this study except taking only few minutes from your time. There will not be any direct payment for the study participants. But the study results would help program managers and policy makers in designing effective strategy to overcome the consequence of undiagnosed diabetes mellitus at all levels.

Any abnormal blood pressure and blood sugar level that need medical attention will be refereed to nearby health facility for treatment. If any problem occurs during drawing blood sample, all necessary measure will be undertaken and the cost will be covered by investigator.

**Confidentiality**: The information you provide will be confidential. There will be no
information that will identify your personal identification. The findings of the study will be general for the study community and will not reflect anything particular of individual persons or housing. The questionnaire was coded to exclude showing names. No reference was made in oral or written reports that could link participants to the research.

**Rights:** Participation in this study is voluntary. You have the right to declare to participate or not to participate in this study.

If you decide to participate, you have the right to withdraw from the study at any time and
this will not label you for any loss of benefits which you otherwise are entitled for. You will not be forced to answer any question that you do not want to answer.

**Contact address**: If you have any questions regarding the study or procedures at any time please contact Mr. Tsegaye Atrese Hal Cell phone- **0949032098/0913427888**

Declaration of informed voluntary consent:

I have read the participants information or it was read to me. I have clearly understood the purpose, the procedures, the risks, the benefits, confidentiality issues, and given the contact address for any questions.

I have been given the opportunity to ask questions for things that may have been unclear. I was informed that I have the right to withdraw from the study at any time or not to answer any question that I do not want to answer.

Therefore, I declare my voluntary consent to participate in this study with my signature as indicated below.
**Signature of the participant_____________________________________________________
Name and Signature of the data collector__________________________________________
Name and signature of data collection supervisor___________________________________
Date: ________/_________/2022G.C**

**Annex 2: Questionnaire (English version).**

| Survey Information | | | |
| --- | --- | --- | --- |
| Location and Date | | Response | Code |
|  | Name of kebele | ___________________ | I1 |
|  | Name of Mender | ___________________ | I2 |
|  | Name of Interviewer | ___________________ | I3 |
|  | Date of completion of the instrument | dd-------/mm-------/year-------- | I4 |

| Participant’s Id Number └─┴─┘ | | | |
| --- | --- | --- | --- |
| Consent, Interview Language and Name | | Response | Code |
|  | Consent has been read and obtained | 1.Yes 2 No If no, stop | I5 |
|  | Interview Language | -------------------- | I6 |
|  | Time of interview (24 hour clock) | hrs└─┴─┘:min└─┴─┘ | I7 |
|  | Contact phone number where possible | ___________________ | I8 |

**Step 1: Socio-Demographic Information**

| **CORE: Demographic Information** | | | |  |
| --- | --- | --- | --- | --- |
| Questions | | | Response | Code |
|  | Sex (Record Male/Female) | | 1. Male 2. Female | C1 |
|  | How old are you? | |  | C2 |
|  | In total, how many years have you spent at school or in full-time study (Excluding pre-school)? | | Years **└─┴─┘** | C3 |
| **EXPANDED :demographic information** | | | | |
|  | What is the highest level of education you have completed? | 1. No formal schooling 2. Less than primary school  3. Primary school completed 4. Secondary school not completed 5. High school completed 6.College/University completed 7. Post graduate degree | | C4 |
|  | What is your ethnic group? | 1. Bench 2. Kaffaa 3. Oromo 4. Amhara 5. Tigre   6. Gurage 7. Silte 8. Other Refused 99 | | C5 |
|  | What is your religion | 1. Protestant 2. Orthodox 3.Muslim 4. Catholic 5. Other | | C6 |
|  | What is your marital status? | 1. Single 2. Married 3. Divorced 4. Widowed | | C7 |
|  | Which of the following best describes your main work status over the past 12 months? | 1. Gov.’t employee 2. NGO employee 3. No-job 4.Merchant 5.Farmer 6. Student 7. Housewife  Refused 99 | | C8 |
|  | How many people older than 18 years, live in your household?(Including yourself) | Number of people └─┴─┘ | | C9 |
|  | What is your gross annual income per household in Ethiopian Birr  (only one, Not all) | Per year------------------------------Go to T1 | | C10a |
|  |  | Per month --------------------------Go to T1 | | C10b |
|  |  | Per week----------------------------Go to T1 | | C10c |
|  |  | Don’t know 77 | |  |
|  |  | Refused 99 | | C10d |

**Step 1 Behavioral Measurements**

| CORE: Tobacco Use | | | | | | | | | | | |
| --- | --- | --- | --- | --- | --- | --- | --- | --- | --- | --- | --- |
| Now I am going to ask you some questions about various health related behaviors. This includes things like smoking, drinking alcohol, eating fruits and vegetables and physical activity. Let's start with tobacco. | | | | | | | | | | | |
|  | Do you currently smoke any tobacco products, like cigarettes or pipes? | | | | | 1. Yes 2. No If no, to T6 | | | | | T1 |
|  | Do you currently smoke tobacco daily? | | | | | 1. Yes 2.No If no, to question T6 | | | | | T2 |
|  | How old were you when you first started smoking daily? | | | | | Age (years) └─┴─┘  Don’t know 77 If known, go to T5a | | | | | T3 |
|  | Do you remember how long ago it was? | | | | | In years └─┴─┘  Or in month └─┴─┘  Or in week └─┴─┘ | | | | | T4a  T4b  T4c |
|  | On average, how many of the following do you smoke each day? For current daily smokers only*)*  *Don’t know 77* | | | | | Manufactured cigarettes **└─┴─┘** | | | | | T5a |
|  |  |  |  |  |  | Hand-rolled cigarettes **└─┴─┘** | | | | | T5b |
|  |  |  |  |  |  | Pipes full of tobacco **└─┴─┘** | | | | | T5c |
|  |  |  |  |  |  | Cigars, cheroots, cigarillos **└─┴─┘** | | | | | T5d |
|  |  |  |  |  |  | Other **└─┴─┘** If Other, go to T5other  else go to T9 | | | | | T5e |
|  |  |  |  |  |  | Other please specify | | | | | T5other |
| **EXPANDED: Tobacco Use** | | | | | | | | | | | |
|  | In the past, did you ever smoke daily? | | | 1. Yes 2.No If no go to T9 | | | | | | | T6 |
|  | How old were you when you stopped smoking daily? Don’t know | | | Age (years)└─┴─┘ If Known, go to T9 | | | | | | | T7 |
|  | Do you remember how long ago it was? (ONLY 1,NOT ALL 3)  Don’t know 77 | | | In Years └─┴─┘ *If Known, go to T9*  OR in Months └─┴─┘ *If Known, go to T9*  OR in Weeks └─┴─┘ | | | | | | | T8a  T8b  T8c |
|  | Do you currently use any  Smokeless tobacco such as *snuff, chewing tobacco, betel*? | | | 1. Yes 2. No If no ,go to T12 | | | | | | | T9 |
|  | Do you currently use smokeless tobacco products daily? | | | 1. Yes 2. No If no, go to T12 | | | | | | | T10 |
|  | On average, how many times a day do you use ….  *(Don't Know 77* | | | Snuff, by mouth └─┴─┘  Snuff, by nose └─┴─┘  Chewing tobacco └─┴─┘  Betel, quid └─┴─┘  Other  *└─┴─*┘  If Other, go to T11 other, else go to T13  Other (specify)└─┴─┴─┴ *Go to T13* | | | | | | | T11a  T11b  T11c  T11d  T11e  T11other |
|  | In the past, did you **ever use** smokeless tobacco such as *snuff, chewing tobacco, or betel* daily? | | | 1. Yes 2. No | | | | | | | T12 |
|  | During the past 7 days, on how many days did someone in your home smoke when you were present? | | | Number of days └─┴─┘  Don’t know 77 | | | | | | | T13 |
|  | During the past 7 days, on how many days did someone smoke in closed areas in your workplace when you were present? *(in* *the building, in a work area or a specific office)* | | | Number of days └─┴─┘  Don't know or don't work in a closed area 77 | | | | | | | T14 |
| **CORE: Alcohol Consumption** | | | | | | | | | | | |
| The next questions ask about the consumption of alcohol. | | | | | | | | | | | |
|  | Have you ever consumed an alcoholic drink such as beer, wine, spirits, fermented cider or | | | | | 1. Yes 2. No If no, go to k1 | | | | | A1a |
|  | Have you consumed an alcoholic drink within the past 12 months? | | | | | 1. Yes 2. No If no, go to k1 | | | | | A1b |
|  | During the past 12 months, how frequently have you had at least one alcoholic drink? | | | | | 1. Daily 2. 5-6 days per week 3. 1-4 days per week 4. 1-3 days per week 5. Less than once per month | | | | | A2 |
|  | Have you consumed an alcoholic drink within the past 30 days? | | | | | 1. Yes 2. No If no, go to k1 | | | | | A3 |
|  | During the past 30 days, on how many occasions have you had at least one alcoholic drink? | | | | | Number**└─┴─┘** Don’t know 77 | | | | | A4 |
|  | During the past 30 days, when you drank alcohol, on average, how many standard alcoholic drinks did you have during one drinking occasion? | | | | | Number**└─┴─┘** Don’t know 77 | | | | | A5 |
|  | During the past 30 days, what was the largest number of standard alcoholic drinks you had on a single occasion, counting all types of alcoholic drinks together? | | | | | Largest number └─┴─┘  Don’t know 77 | | | | | A6 |
|  | During the past 30 days, how many times did you have:  for men five or more  For women: four or more standard alcoholic drinks in a single drinking occasion? | | | | | Number└─┴─┘ Don’t know 77 | | | | | A7 |
| **EXPANDED: Alcohol Consumption** | | | | | | | | | | | |
|  | During the past 30 days, when you consumed an alcoholic drink, how often was it with meals?  (Please do not count snacks.) | | | | | 1. Usually with meals 2. Sometimes with meals 3. Rarely with meals 4. Never with meals | | | | | A8 |
|  | During each of the past 7 days, how many standard drinks of any alcoholic drink did you have each day?  *Don't know 77* | | | | | Monday └─┴─┘ | | | | | A9a |
|  |  |  |  |  |  | Tuesday └─┴─┘ | | | | | A9b |
|  |  |  |  |  |  | Wednesday └─┴─┘ | | | | | A9c |
|  |  |  |  |  |  | Thursday └─┴─┘ | | | | | A8d |
|  |  |  |  |  |  | Friday └─┴─┘ | | | | | A9e |
|  |  |  |  |  |  | Saturday └─┴─┘ | | | | | A9f |
|  |  |  |  |  |  | Sunday └─┴─┘ | | | | | A9g |
| **Khat chewing** | | | | | | | | | | | |
|  | Have you ever chew khat? | | | | | 1.Yes 2.No , if no go to D1 | | | | | K1 |
|  | Do you currently chew khat? | | | | | 1.Yes 2.No | | | | | K2 |
|  | During the past 12 month how frequently did you chew khat? | | | | | 1.daily  2. 5-6 per week  3. 3-4 per week  4. 1-2 per week  5. 1-3 per month  6. less than 1 per month | | | | | K3 |
|  | How old was you when you first started khat chewing? | | | | | ----------------------------------- | | | | | K4 |
|  | Do you remember how long ago it was? | | | | | 1.year ---------------------------  2.month ------------------------  3.week-------------------------- | | | | | K5a  K5b  K5c |
|  | On average how many bundles of khat do you chew each day or week? | | | | | ----------------------------------- | | | | | K6 |
|  | During the last 12 month have you tried to stop chewing khat? | | | | | 1.Yes 2.No | | | | | K7 |
| **CORE: Diet** | | | | | | | | | | | |
| The next questions ask about the fruits and vegetables that you usually eat. I have a nutrition card here that shows you some examples of local fruits and vegetables. Each picture represents the size of a serving. As you answer these questions please think of a typical week in the last year. | | | | | | | | | | | |
|  | In a typical week, on how many days do you eat fruit? | | | | | Number └─┴─┘ If zero, go to D3 Don’t know 77 | | | | | D1 |
|  | How many servings of fruit do you eat on one of those days? | | | | | Number of servings └─┴─┘  Don’t know 77 | | | | | D2 |
|  | In a typical week, on how many days do you eat vegetables? | | | | | Number days └─┴─┘ If zero, go to D5 Don’t know 77 | | | | | D3 |
|  | How many servings of vegetables do you eat on one of those days? | | | | | Number servings └─┴─┘  Don’t know 77 | | | | | D4 |
| **EXPANDED: Diet** | | | | | | | | | | | |
|  | What type of oil or fat is most often used for meal preparation in your household?  Don’t know 77 | | | | 1. Vegetable oil 5. Garligi 2. Butter 6. None in particular 3. Shano 7. Not different 4. Margarine 8. Other | | | | | | D5 |
|  |  |  |  |  | Other └─┴─┴─┴─┴─┴─┴─┘ | | | | | | D5others |
|  | On average, how many meals per week do you eat that were not prepared at a home? | | | | Number servings └─┴─┘  Don’t know 77 | | | | | | D6 |
| CORE: Physical Activity | | | | | | | | | | | |
| Next I am going to ask you about the time you spend doing different types of physical activity in a typical week. Please answer these questions even if you do not consider yourself to be a physically active person. Think first about the time you spend doing work. Think of work as the things that you have to do such as paid or unpaid work, study/training, household chores, harvesting food/crops, fishing or hunting for food, seeking employment. | | | | | | | | | | | |
|  | Does your work involve vigorous-intensity activity that causes large increases in breathing or heart rate for at least 10 minutes continuously? (*Carrying or lifting heavy loads, digging or construction work)* | | | | | | | 1. Yes 2. No If no, go to P4 | | | P1 |
|  | In a typical week, on how many days do you do vigorous-intensity activities as part of your work? | | | | | | | Number of days**└─┘** | | | P2 |
|  | How much time do you spend doing vigorous-intensity activities at work on a typical day? Think of one day you can recall easily.  Consider only those activities undertaken continuously for 10 minutes or more. Probe very high responses (over 4 hrs) to verify. | | | | | | | Hours: minutes  └─┴─┘:└─┴─┘  hrs mins | | | P3  (a-b) |
|  | Does your work involve moderate-intensity activity that causes small increases in breathing or heart rate such as brisk walking for at least 10 minutes continuously?  **[**or carrying light loads] | | | | | | | 1. Yes 2. No If no, go to P7 | | | P4 |
|  | In a typical week, on how many days do you do moderate intensity activities as part of your work? | | | | | | | Number of days └─┘ | | | P5 |
|  | How much time do you spend doing moderate-intensity activities at work on a typical day? Probe very high responses (over 4 hrs) to verify Travel to and from places to place | | | | | | | Hours minutes └─┴─┘: └─┴─┘ | | | P6  (a-b) |
| The next questions exclude the physical activities at work that you have already mentioned. Now I would like to ask you about the usual way you travel to and from places. For example to work, for shopping, to market, to place of worship. | | | | | | | | | | | |
|  | Do you walk or use a bicycle (pedal cycle) for at least 10 minutes continuously to get to and from places? | | | | | 1. Yes 2. No If no, go to P10 | | | | P7 | |
|  | In a typical week, on how many days do you walk or bicycle for at least 10 minutes continuously to get to and from places? | | | | | Number of days└─┘ | | | | P8 | |
|  | How much time do you spend walking or bicycling for travel on a typical day? *Probe very high responses* />4hr | | | | | Hours : minutes └─┴─┘: └─┴─┘  hrs mins | | | | P9  (a-b) | |
| **Recreational activities** | | | | | | | | | | | |
| The next questions exclude the work and transport activities that you have already mentioned. Now I would like to ask you about sports, fitness and recreational activities. | | | | | | | | | | | |
|  | Do you do any vigorous-intensity sports, fitness or recreational (leisure) activities that cause large increases in breathing or heart rate like [running or football] for at least 10 minutes continuously? | | | | | 1. Yes 2. No If no, go to 13 | | | | P10 | |
|  | In a typical week, on how many days do you do vigorous-intensity sports, fitness or recreational (leisure) activities? | | | | | Number of days └─┘ | | | | P11 | |
|  | How much time do you spend doing vigorous-intensity sports, fitness or recreational activities on a typical day?  (*Probe very high responses (over 4 hrs))* | | | | | Hours : minutes└─┴─┘: └─┴─┘  hrs mins | | | | P12  (a-b) | |
|  | Do you do any moderate-intensity sports, fitness or recreational activities that cause a small increase in breathing or heart rate such as brisk walking**,** (cycling, swimming, and volleyball) for at least 10 minutes continuously? | | | | | 1. Yes 2. No If no go to P16 | | | | P13 | |
|  | In a typical week, on how many days do you do moderate-intensity sports, fitness or recreational activities? | | | | | Number of days └─┘ | | | | P14 | |
|  | How much time do you spend doing moderate-intensity sports, fitness or recreational activities on a typical day? | | | | | Hours: minutes └─┴─┘: └─┴─┘  hrs mins | | | | P15  (a-b) | |
| **EXPANDED: Physical Activity** | | | | | | | | | | | |
| **Sedentary behavior** | | | | | | | | | | | |
| The following question is about sitting or reclining at work, at home, getting to and from places, or with friends including time spent sitting at a desk, sitting with friends, traveling in car, bus, train, reading, playing cards or watching television, but do not include time spent sleeping. | | | | | | | | | | | |
|  | | How much time do you usually spend sitting or reclining on a typical day?  (Do not include time spent sleeping) | | | | Hours : minutes └─┴─┘: └─┴─┘  hrs mins | | | | P16  (a-b) | |
| **CORE: History of Raised Blood Pressure** | | | | | | | | | | | |
|  | | Have you ever had your blood pressure measured by a doctor or other health worker? | | | | 1. Yes 2. No If no, go to H6 | | | | H1 | |
|  | | Have you ever been told by a doctor or other health worker that you have raised blood pressure or hypertension? | | | | 1. Yes 2. No If no, go to H6 | | | | H2a | |
|  | | Have you been told in the past 12 months? | | | | 1. Yes 2. No | | | | H2b | |
| **EXPANDED: History of Raised Blood Pressure** | | | | | | | | | | | |
|  | | Are you currently receiving any of the following treatments/advice for high blood pressure prescribed by a doctor or other health worker? | | | | | | | | | |
|  |  | 1. Drugs (medication) that you have taken in the past two weeks | | | | | | | | H3a | |
|  |  | 1. Advice to reduce salt intake | | | | | | | | H3b | |
|  |  | 1. Advice or treatment to lose weight | | | | | | | | H3c | |
|  |  | 1. Advice or treatment to stop smoking | | | | | | | | H3d | |
|  |  | 1. Advice to start or do more exercise | | | | | | | | H3e | |
|  | | Have you ever seen a traditional healer for raised blood pressure or hypertension? | 1. Yes 2. No | | | | | | | H4 | |
|  | | Are you currently taking any herbal or traditional remedy for your raised blood pressure? | 1. Yes 2. No | | | | | | | H5 | |
| **CORE: History of Diabetes** | | | | | | | | | | | |
|  | | Have you ever had your blood sugar measured by a doctor or other health worker? | | | | | 1. Yes 2. No If no, go to M1 | | | H6 | |
|  | | Have you ever been told by a doctor or other health worker that you have raised blood sugar or diabetes? | | | | | 1. Yes 2. No If no, go to M1 | | | H7a | |
|  | | Have you been told in the past 12 months? | | | | | 1. Yes 2. No | | | H7b | |
| **EXPANDED: History of Diabetes** | | | | | | | | | | | |
|  | | Are you currently receiving any of the following treatments/advice for diabetes prescribed by a doctor or other health worker? | | | | | | | |  | |
|  |  | Insulin | | | | | | | 1. Yes 2.No | H8a | |
|  |  | Drugs (medication) that you have taken in the past two weeks | | | | | | | 1. Yes 2.No | H8b | |
|  |  | Special prescribed diet | | | | | | | 1. Yes 2.No | H8c | |
|  |  | Advice or treatment to lose weight | | | | | | | 1. Yes 2.No | H8d | |
|  |  | Advice or treatment to stop smoking | | | | | | | 1. Yes 2. No | H8e | |
|  |  | Advice to start or do more exercise | | | | | | | 1. Yes 2.No | H8f | |
|  | | Have you ever seen a traditional healer for diabetes or raised blood sugar? | | | | | | | 1. Yes 2. No | H9 | |
|  | | Are you currently taking any herbal or traditional remedy for your diabetes? | | | | | | | 1. Yes 2. No | H10 | |
|  | | During pregnancy did your mother had gestational diabetes? | | | | | | | 1.Yes  2. No | H11g | |
|  | | Is there any family member or relatives (father, mother, sibling, grand family?)Who are diagnosed for diabetes or have history of diabetes mellitus? | | | | | | | 1. Yes 2. No | H11 | |
| **Step 2 Physical Measurements** | | | | | | | | | | | |
| **CORE: Height and Weight** | | | | | | | | | | | |
|  | | Interviewer ID( for Ht,wt &WC) | └─┴─┴─┘ | | | | | | | M1 | |
|  | | Device IDs for height and weight | Height └─┴─┘  Weight └─┴─┘ | | | | | | | M2a  M2b | |
|  | | Height**(**Record participant's height in cm) | In Centimeters (cm) └─┴─┴─┘.└─┘ | | | | | | | M3 | |
|  | | Weight (If too large for scale, code 666.6 | In Kilograms (kg) └─┴─┴─┘.└─┘ | | | | | | | M4 | |
|  | | For women: Are you pregnant? | 1. Yes If Yes, go to M 8 2. No | | | | | | | M5 | |
| **CORE: Waist** | | | | | | | | | | | |
|  | | Device ID for waist | └─┴─┘ | | | | | | | M6 | |
|  | | Waist circumference | In Centimeters (cm) ---------------- | | | | | | | M7 | |
| **CORE: Blood Pressure** | | | | | | | | | | | |
|  | | Interviewer ID | └─┴─┴─┘ | | | | | | | M8 | |
|  | | Device ID for blood pressure | └─┴─┘ | | | | | | | M9 | |
|  | | Cuff size used | 1. Small 2. Medium 3. Large | | | | | | | M10 | |
|  | | Reading 1 | Systolic ( mmHg) └─┴─┴─┘  Diastolic (mmHg) └─┴─┴─┘ | | | | | | | M11a  M11b | |
|  | | Reading 2 (Record first measurement after the participant has rested for 15 minutes and wait 3 minutes before taking 3^rd^ measurement) | Systolic ( mmHg) └─┴─┴─┘  Diastolic (mmHg) └─┴─┴─┘ | | | | | | | M12a  M12b | |
|  | | Reading 3  (Record first measurement after the participant has rested for 15 minutes). | Systolic ( mmHg) └─┴─┴─┘  Diastolic (mmHg) └─┴─┴─┘ | | | | | | | M13a  M13b | |
|  | | During the past two weeks, have you been treated for raised blood pressure with drugs (medication) prescribed by a doctor or other health worker? | 1. Yes 2. No | | | | | | | M14 | |
|  | | Is there any family member, father ,mother, grand parent or siblings with diabetes mellitus | 1.yes 2.no | | | | | | | M 15 | |
| **EXPANDED: Hip Circumference and Heart Rate** | | | | | | | | | | | |
|  | | Hip circumference | In Centimeters (cm) └─┴─┴─┘.└─┘ | | | | | | | M16 | |
|  | | Heart Rate (Record the three heart rate readings) | | | | | | | | | |
|  |  | Reading 1 | Beats per minute └─┴─┴─┘ | | | | | | | M17a | |
|  |  | Reading 2 | Beats per minute └─┴─┴─┘ | | | | | | | M17b | |
|  |  | Reading 3 | Beats per minute └─┴─┴─┘ | | | | | | | M17c | |
| **Step 3 Biochemical Measurements** | | | | | | | | | | | |
| **CORE: Blood Glucose** | | | | | | | | | | | |
|  | | During the past 12 hours have you had anything to eat or drink, other than water? | | | | | 1. Yes 2. No | | | B1 | |
|  | | Technician ID | | | | | └─┴─┴─┘ | | | B2 | |
|  | | Device ID | | | | | └─┴─┘ | | | B3 | |
|  | | Time of day blood specimen taken (24 hour clock) | | | | | Hours -----: minutes ----- | | | B4 | |
|  | | Fasting blood glucose | | | | | Mg /dl └─┴─┘. └─┴─┘ | | | B5 | |
|  | | Today, have you taken insulin or other drugs (medication) that have been prescribed by a doctor or other health worker for raised blood glucose? | | | | | 1. Yes 2. No | | | B6 | |
