# Supplementary material for: Prevalence of undiagnosed diabetes mellitus and associated factors among adult residents of Mizan Aman town, Southwest Ethiopia: Community-based cross-sectional study
Source: PLoS One. 2024 May 7;19(5):e0302167. doi: 10.1371/journal.pone.0302167 (PMC11075875; doi:10.1371/journal.pone.0302167)
Supplement: Attachment — Submitted filename: Comment.docx [file pone.0302167.s005.docx]

**Prevalence of undiagnosed diabetes mellitus and associated factors among adult residents of Mizan Aman town, Southwest Ethiopia: Community-based cross-sectional study**

Thank you very much for giving me the opportunity to review this well written Manuscript

From the introduction part of the abstract section: You should show why you are going to conduct this study? Is it a public health concern?

What is WHO STEP wise method? Is it a tool? If so, determine its validity and reliability? The information present in the abstract should be present the main document. But this information/statement is not found in the main document.

Why did you use fasting blood glucose level to determine whether the individual has diabetes mellitus or not? Why did you fail to use either random blood glucose or OGTT?

Did you think that only single measurement of blood glucose is sufficient to declare whether the patient has diabetes mellitus or not? How do you exclude those risk factors which may elevate blood glucose level?

Did you think that only single measurement of blood pressure is sufficient to declare whether the patient has hypertension or not? How do you exclude those risk factors which may elevate blood pressure level?

Could you explain the entire procedure to measure random blood glucose level? How do you know, if the person eats breakfast and he said that he is fasting for more than 8 hour? How do you detect such types of error/bias? How do you detect measurement error? Do you know the sensitivity and specificity of the measurement tool?

Why do you focus on the prevalence of undiagnosed DM among adult people? Did you think that, it is not common in pediatrics age group? Or DM is not a concern for pediatrics population?

As you stated in the introduction section, despite different strategies were undertaken to reduce the incidence of DM, the majority of individuals come to healthcare after the disease has been progressed and multiple organ damage has occurred. So, it is better to study why people fail to measure/to be measured their blood glucose level regularly? As per my understanding, the effect of undiagnosed DM is not mentioned.

Why you are going to conduct this study, since it is studied and published in the different regions and corners of the country? You just tried to justify under the last paragraph of the introduction section but it is not persuasive as intended.

What is your inclusion criterion?

Why did you exclude pregnant women and adults who were taking beta blockers within two weeks of data collection?

Why did you use 3% margin of error, and 1.5 design effect?

The sampling procedure is not well explained. So it needs revision and it is recommended to support using diagrammatic presentation. In the case of households with no eligible participant at the time of visit, the next nearby household was selected as a substitute why you fail to revisit?

The tool is adapted from Ethiopia's steps report on risk factors for non-communicable diseases and the prevalence of selected NCDs (25)……it is not similar with the idea stated under reference 25. So. It needs revision. Similarly see reference number 28

Could you explain about the specificity and sensitivity of CareSens TM N Eco digital glucometer?

Why did you select those variables with a p-value < 0.25 in bi-variable logistic regression transferred to multi-variable logistic regression model to adjust for confounder effect? What is your evidence and justification???

What is the declaration of Helsinki? How did you get blood sample? Did the study participants were volunteer? Could you tell me any event you experienced while collecting blood from the people? Did they ask any compensation for the harm? Please explain each ethical standard.

How do you classify monthly income in to these categories? Did you have any evidence? If so what is your evidence?

In bi-variable analysis, 12 variables were identified as statistically significant variable……..it is not necessary to mention the number rather it is enough to mention the variable solely.

Why did you fail to interpret each and every variable’s which had statistically significant association with the outcome variable? Please interpret each variable

Some terminology needs operational definition. Eg sedentary life

In the discussion section, you write reference number rather than writing the study setting/area. Please write the specific area/town/country and some justification given for the possible association is not persuasive. So it needs revision.

Your strength of the study is not ideal
