# Supplementary material for: Prevalence of undiagnosed diabetes mellitus and associated factors among adult residents of Mizan Aman town, Southwest Ethiopia: Community-based cross-sectional study
Source: PLoS One. 2024 May 7;19(5):e0302167. doi: 10.1371/journal.pone.0302167 (PMC11075875; doi:10.1371/journal.pone.0302167)
Supplement: Attachment — Submitted filename: Authors response to reviewers.docx [file pone.0302167.s006.docx]

**Author's Responses to reviewers**

**Reviewer #1:**

1. From the introduction part of the abstract section: You should show why you are going to conduct this study? Is it a public health concern?

**Response:** We greatly appreciate the reviewer’s positive comments. We have now included a statement that indicates the issue as a public health concern and the associated gaps. (See page 1; line number 12-15 of the manuscript.).

2. What is WHO STEP wise method? Is it a tool? If so, determine its validity and reliability? The information present in the abstract should be present the main document. But this information/statement is not found in the main document.

**Response:** We greatly appreciate the reviewer’s positive comments. The method was standardized by WHO and implemented in other researches nationally such as by EPHI. And we make the information in abstract and the main document consistent.

3. Why did you use fasting blood glucose level to determine whether the individual has diabetes mellitus or not? Why did you fail to use either random blood glucose or OGTT?

**Response:** We are very grateful for the reviewer's remarks. This is because glucose levels are measured under consistent conditions, minimizing the impact of recent food intake or other factors that can affect glucose levels. Furthermore, this is the recommendation of WHO for a research data collection on blood glucose. (See page 3-6-7 of reference: *World Health Organization. The WHO STEPwise approach to noncommunicable disease risk factor surveillance. 2017.)*

4. Did you think that only single measurement of blood glucose is sufficient to declare whether the patient has diabetes mellitus or not? How do you exclude those risk factors which may elevate blood glucose level?

**Response:** We greatly appreciate the reviewer’s concern. As we have stated before, we used the WHO STEP wise approach to collect data. The guideline recommends to take a fasting blood glucose level only once. Moreover, since we are taking FBG, factors which may elevate blood glucose level are controlled. (See page 3-6-7 of reference: *World Health Organization. The WHO STEPwise approach to noncommunicable disease risk factor surveillance. 2017.)*

5. Did you think that only single measurement of blood pressure is sufficient to declare whether the patient has hypertension or not? How do you exclude those risk factors which may elevate blood pressure level?

**Response:** We greatly appreciate the reviewer’s important remark. You are correct, in a clinical diagnosis of high BP, an individual should have elevated BP on at least two visits, on different days. However, our study is not a clinical diagnosis. In research settings, the measurement criteria vary based on standard guidelines for specific research. Accordingly, as we are using the WHO STEPwise approach, our measurement method is in line with the guideline. Furthermore, since most research is conducted using this approach, it is preferable to use the same approach to ensure comparability.

We appreciate your feedback on the second question, and we have made updates based on your recommendation. (See 7, line number 187-193).

6. Could you explain the entire procedure to measure random blood glucose level? How do you know, if the person eats breakfast and he said that he is fasting for more than 8 hour? How do you detect such types of error/bias? How do you detect measurement error? Do you know the sensitivity and specificity of the measurement tool?

**Response:** We greatly appreciate the reviewer’s positive comments.

Firstly, we did not utilize a random blood glucose test as it is not recommended by the WHO for research purposes. Rather we used fasting blood glucose. Additionally, to prevent inappropriate responses from study participants, we explicitly explained the study's purpose, the significance of their genuine responses to the findings, and how the results would be used within the community and the nation. Consequently, by ensuring that study participants comprehensively understood the study's purpose, we minimized potential biases.

7. Why do you focus on the prevalence of undiagnosed DM among adult people? Did you think that, it is not common in paediatrics age group? Or DM is not a concern for paediatrics population?

**Response:** We greatly appreciate the reviewer’s positive comments. Certainly, nowadays, the prevalence of childhood DM is rising both globally and regionally. However, while it is crucial to examine childhood DM, we believe this group shall be investigated in a separate study due to their distinct characteristics and causes.

8. As you stated in the introduction section, despite different strategies were undertaken to reduce the incidence of DM, the majority of individuals come to healthcare after the disease has been progressed and multiple organ damage has occurred. So, it is better to study why people fail to measure/to be measured their blood glucose level regularly? As per my understanding, the effect of undiagnosed DM is not mentioned.

**Response:** We greatly appreciate the reviewer’s positive comments.

- You are right, our study could be one of the studies to answer this question basically the factors and the prevalence. Moreover, to answer the question why, qualitative studies should be conducted and we will consider a qualitative study for our next work.
- If we clearly understand your question, we think we have indicated the impact of UDM such as multi organ damage.

9. Why you are going to conduct this study, since it is studied and published in the different regions and corners of the country? You just tried to justify under the last paragraph of the introduction section but it is not persuasive as intended.

**Response:** We greatly appreciate the reviewer’s positive comments.

Based on existing research, the prevalence of undiagnosed DM in Ethiopia ranges from 2.3% to 11.5%, indicating a significant difference that may be attributed to socio-economic, cultural, and lifestyle variations across different study areas. This variability underscores the need for region-specific studies, as the problem varies throughout the nation. Therefore, conducting a specific study in southwest Ethiopia offers valuable insights that may differ from findings in other parts of the country, and is crucial for addressing the unique healthcare needs of the population in southwest Ethiopia.

10. What is your inclusion criterion?

**Response:** We greatly appreciate the reviewer’s positive comments.

- Adults over 18 years old, who have lived in the study area for 6 months or more and have not been previously diagnosed with diabetes mellitus were included in the study. This in also included in the revised manuscript. Please see page 5, line number 122-126.

11. Why did you exclude pregnant women and adults who were taking beta blockers within two weeks of data collection?

**Response:** We greatly appreciate the reviewer’s positive comments.

- Pregnancy has a significant impact on blood glucose measurement, primarily due to the hormonal changes that occur during this period, which can affect the accuracy and interpretation of blood glucose readings.
- Beta-blockers are drugs used to treat high BP and heart conditions. They have the side effect of raising blood glucose levels, which can impact the measurement and the study. Therefore, we have excluded both groups to minimize bias.

12. Why did you use 3% margin of error, and 1.5 design effect?

**Response:** We greatly appreciate the reviewer’s positive comments.

- A 3% margin of error was employed to increase the precision and accuracy of the obtained results. As the margin of error decreases, the sample size will increase, which is recommended when the assumed proportion to calculate sample size is low (in our case, the assumed proportion is 10.2%).
- Because we employed a multi-stage sampling method, we need to account for the design effect when calculating the sample size. While the typical design effect number is 2, which can significantly increase the sample size, we opted for 1.5 due to the resource constraints in our country. This decision is also backed by numerous studies in Ethiopia that have utilized multi-stage sampling.

13. The sampling procedure is not well explained. So it needs revision and it is recommended to support using diagrammatic presentation. In the case of households with no eligible participant at the time of visit, the next nearby household was selected as a substitute why you fail to revisit?

**Response:** We are very thankful for the reviewer's favourable remarks. The procedures have now been clearly explained, and diagrammatic presentations have been incorporated. Please see page 5 and 6.

14. The tool is adapted from Ethiopia's steps report on risk factors for non-communicable diseases and the prevalence of selected NCDs (25) …… it is not similar with the idea stated under reference 25. So, it needs revision. Similarly see reference number 28

**Response:** We greatly appreciate the reviewer’s positive comments. Now we have corrected the references.

15. Could you explain about the specificity and sensitivity of Care Sens TM N Eco digital glucometer?

**Response:** We greatly appreciate the reviewer’s positive comments. **System accuracy** of ‘Care Sens TM N Eco digital glucometer’ for glucose concentrations is between 36.4 mg/dL and 471.1 mg/dL, (within ±15mg/dL and ±15%) is **99%,** which is comparable with other standard devices. Please also kindly see device information from the following link:

<https://i-sens.com/pdf/PGUAA-0000047-REV0-CS-N-Eco-M-Manual-CE-EN.pdf>

16. Why did you select those variables with a p-value < 0.25 in bi-variable logistic regression transferred to multi-variable logistic regression model to adjust for confounder effect? What is your evidence and justification???

**Response:** We greatly appreciate the reviewer’s positive comments.

The first reason is that it is a fairly conservative threshold, which is unlikely to include variables that are not truly linked to the outcome variable. The second reason is that it is a widely accepted threshold in many different fields of research. Therefore, using the same method is preferable for ensuring comparability of our study with existing research.

17. What is the declaration of Helsinki? How did you get blood sample? Did the study participants were volunteer? Could you tell me any event you experienced while collecting blood from the people? Did they ask any compensation for the harm? Please explain each ethical standard.

**Response:** We greatly appreciate the reviewer’s positive comments.

The Declaration of Helsinki comprises ethical guidelines for medical research involving human subjects. As mentioned in the ethical considerations of the methodology section, we adhered to all these principles. The participants volunteered, and we encountered no refusals to provide blood samples. They did not request any compensation; instead, they were pleased to receive their health status information free of charge.

18. How do you classify monthly income in to these categories? Did you have any evidence? If so what is your evidence?

**Response:** We greatly appreciate the reviewer’s positive comments. This is because the majority of previous studies have employed the same income classification. Utilizing consistent classification with prior studies improves comparability across research. Similar categories have been utilized in another studies.

19. In bi-variable analysis, 12 variables were identified as statistically significant variable……..it is not necessary to mention the number rather it is enough to mention the variable solely.

**Response:** We greatly appreciate the reviewer’s positive comments. Now we have excluded the number and mentioned the variables. Please see page 14, line number 297-315.

20. Why did you fail to interpret each and every variable’s which had statistically significant association with the outcome variable? Please interpret each variable.

**Response:** We greatly appreciate the reviewer’s positive comments. Now all significant variables are interpreted. Please see page 14, line number 297-315.

21. Some terminology needs operational definition. Eg sedentary life.

**Response:** We greatly appreciate the reviewer’s positive comments.

Sedentary behaviours are activities that involve very little physical movement such as sitting at home traveling in a car, watching television, using social media, and sitting at office work. As indicated in the result part it is analysed as sedentary behaviour for < 4hr per day and ≥ 4 hour per day. Now this and other terms that need operational definition are included in the manuscript. Please see page 8, line number 223.

22. In the discussion section, you write reference number rather than writing the study setting/area. Please write the specific area/town/country and some justification given for the possible association is not persuasive. So it needs revision.

**Response:** We greatly appreciate the reviewer’s positive comments. We have now indicated the exact places of the studies.

23. Your strength of the study is not ideal.

**Response:** We greatly appreciate the reviewer’s positive comments. We have improved indicated part.

**Reviewer #2:**

1. There are grammatical and syntax errors throughout the manuscript. The manuscript needs extensive review by the author and should also be checked by a professional speaker of English.

Response: We greatly appreciate the reviewer’s positive comments. We attempted to address grammatical mistakes by submitting the manuscript for review by senior individuals.

2. Please write abbreviations in full at the first time of use.

Response: We greatly appreciate the reviewer’s positive comments. We have attempted to deal with this particular comment. If the same issues arise again, it would be helpful to specify the exact points.

3. The objectives and rationale of the study are not clearly stated. Why not include impaired fasting sugar level, or pre-DM, and its risk factors in your study? Previously, almost similar studies had been conducted in the same study area, so what is the significance of doing this study?

**Response:** It is crucial to investigate pre-diabetes as well. Additionally, other related areas such as diabetes mellitus and childhood diabetes could be explored concurrently. Nonetheless, based on the authors' understanding, narrowing down the topic is recommended to enhance clarity and simplicity to readers, offer specific recommendations, and improve comprehension.

Abstract:

4. Background: In the background part, include the problem or identified gap that you want to fill.

**Response:** We greatly appreciate the reviewer’s positive comments. We have improved the background based on your feedback. Please see page 1, line number 12-15.

5. Methods: Please include the laboratory method that you used to determine the fasting blood sugar level briefly.

**Response:** We greatly appreciate the reviewer’s positive comments. We have included the following statement with in the method part of the abstract "The fasting blood glucose level was measured after an 8-hour gap following a meal, using a digital glucometer to analyse a blood sample." Please see page 1, line number 23-25.

6. Avoid abbreviations from the abstract.

**Response:** We greatly appreciate the reviewer’s positive comments. We have now avoided abbreviations.

7. Introduction: Add information about the complications and risk factors of DM. Some literature is not up-to-date and needs to be reviewed properly.

**Response:** We greatly appreciate the reviewer’s positive comments. Now we have addressed comments.

Methods and materials:

8. The sampling technique is not well stated. How many households were in each cluster? How was the selection made? Please include the detailed sampling frame.

**Response:** We greatly appreciate the reviewer’s positive comments. Now we have improved the sampling technique as per your feedback. Please see page 5 and 6.

9. How can you be sure that patients were fasting overnight? Is there any system used to convince them and trust them?

**Response:** We appreciate the positive feedback from the reviewer. To ensure that patients were fasting overnight, we took several steps to encourage their compliance and build trust. Firstly, we provided a clear explanation of the study's objectives and its significance in supporting the healthcare system. This approach aimed to motivate participants to provide accurate information or honest verbal reports.

Moreover, we conducted blood glucose measurements early in the morning following an overnight fast and asked participants about their recent food intake. If they had not eaten, we proceeded with measuring their blood glucose levels. For those who had already eaten, we informed them that we would return early the next morning and kindly requested them to refrain from eating until we could obtain a blood sample. Fortunately, due to the participants' interest in knowing their health status, they were cooperative throughout the process, and we did not encounter the challenges we had anticipated theoretically.

10. Add relevant citations for important statements.

**Response:** We greatly appreciate the reviewer’s positive comments. We have improved our citation. But, if there are still similar issues, let us know specific areas of the comment.

11. Quality control measures are inadequate. What quality control measures were you using for the CareSens^TM^ N Eco digital glucometer to ensure the method was safe?

**Response:** We greatly appreciate the reviewer’s positive comments. The first quality control measure was assured using CareSens Control Solutions (control A and/or B) test. The CareSens Control Solution contains a known amount of glucose and is used to check that the meter and the test strips are working properly. The details for this method are available on page number 15 of the devices system information available at: <https://i-sens.com/pdf/PGUAA-0000047-REV0-CS-N-Eco-M-Manual-CE-EN.pdf>

Furthermore, data collectors are adequately trained by laboratory technologist on how to handle and measure the blood sample.

12. Please operationally define physical activity, sedentary life, hypertension, undiagnosed DM, and so on.

**Response:** We greatly appreciate the reviewer’s positive comments. Terms that need operational definition are now defined. Please see page 8, line number 223.

13. Ethical consideration: What was done for those with raised blood pressure?

**Response:** We greatly appreciate the reviewer’s positive comments. Now we have included what was done for those who have high blood pressure. Please see page 10, line number 258.

Results:

14. The presentation of the results is poor in terms of sentence form, coherence, outcome measurement, and lots of grammatical errors. The authors are unable to provide the most important result in a short and precise manner. Furthermore, the results are simply summarized in the tables without sufficient explanation. In general, the results section needs major modification.

**Response:** We greatly appreciate the reviewer’s positive comments. We have updated the result part accordingly.

15. The authors did not include some important variables, such as lipid profile, in their study.

**Response:** We greatly appreciate the reviewer’s positive comments. Yes, we didn’t include lipid profile though it is important variable. This is because of resource limitation. And now we have included this under limitation of the study. Please see page 20, line number 389.

16. Diagrams and figures are missing. Add figures and diagrams.

**Response:** We greatly appreciate the reviewer’s positive comments. Now we have included the graph of blood glucose level. Please see page 14.

17. The study has many more limitations, but the authors did not include them as the limitations of the study.

**Response:** We greatly appreciate the reviewer’s positive comments. We have revised it as your suggestion.

**Reviewer #3: General comment**

1. The author tried to estimate the prevalence of undiagnosed DM and its predictors among residents of Mizan Aman town. However, the predictive variables such as hypertension, family History, sedentary life, and physical activity are the very well-known risk factors for DM. So what value this research could add to the scientific world. This is the major gap of the study.

**Response:** we could have been happy if we came up with new findings but, it should not be discouraged due to similar finding/ known risk factor for DM because it may suggest some clue for causal association with limitation. Our result has also reported the prevalence of the outcome specific to the locality.

Introduction

2. Most of the sentences in this section are not paraphrased by the authors own word. For instance the first sentence is directly copied from somewhere. So the authors have to re-write the sentences by their own word.

**Response:** Thank you for the comment. Comment accepted and addressed.

Methods

3. Why adults taking beta blockers are excluded from the study?

**Response:** We greatly thank for the comment. Beta blockers have the side effect of raising blood glucose levels, which can impact the measurement and the study. Therefore, we have excluded them to minimize bias.

4. The tools the author used for dietary questions should be described in detail.

**Response:** We greatly appreciate the reviewer’s positive comments and we addressed them now. Please see page 7, line number 182-186.

5. Some terms like ever drinker/chewer, current chewer/drinker, and physical activity should be operationally defined.

**Response:** comment accepted and operationalized. Please see page 8.

Result

6. In table 2, there is a variable “history of gestational diabetes”. I think this variable should be only for female participants. But the authors used it for both males and females. How?

**Response:** It was a mistake and corrected.

7. In table 3, the P-value for COR should be stated.

**Response:** We accept your comment, but because table size we could not able to include p-value for COR. preferably we placed p-value for AOR.

Discussion and conclusion

8. Being community based study by itself cannot be the strength of your study. There are an ample community based studies conducted on undiagnosed DM among residents of Ethiopia.

**Response:** We greatly appreciate the reviewer’s positive comments. Your comments are considered.

9. The conclusion and recommendation part is very weak. There is no direction toward future studies, emphasis on what needs to be done, or what is missing.

**Response:** We greatly appreciate the reviewer’s positive comments. Now we have amended as per the feedback.
